# Research priorities for therapeutic plasma exchange in critically ill patients

**DOI:** 10.1186/s40635-023-00510-w

**Published:** 2023-05-08

**Authors:** Sascha David, Lene Russell, Pedro Castro, Andry van de Louw, Lara Zafrani, Tasneem Pirani, Nathan D. Nielsen, Eric Mariotte, Bruno L. Ferreyro, Jan T. Kielstein, Luca Montini, Anne C. Brignier, Matthias Kochanek, Joan Cid, Chiara Robba, Ignacio Martin-Loeches, Marlies Ostermann, Nicole P. Juffermans, for the Nine-I investigators

**Affiliations:** 1 Institute of Intensive Care Medicine, University Hospital Zurich, Zurich, Switzerland; 2 Department of Nephrology and Hypertension, Hannover Medical School, Hannover, Germany; 3 Department of Intensive Care, Copenhagen University Hospital Gentofte, Copenhagen, Denmark; 4 Department of Clinical Medicine, University of Copenhagen, Copenhagen, Denmark; 5 Medical Intensive Care Unit, Hospital Clínic of Barcelona, IDIBAPS, University of Barcelona, Barcelona, Spain; 6 Medical Intensive Care Unit, Penn State Health Hershey Medical Center, Hershey, PA USA; 7 Medical Intensive Care Unit, Saint-Louis Hospital, AP-HP, University of Paris Cité, Paris, France; 8 King’s College Hospital, General and Liver Intensive Care, London, UK; 9 Division of Pulmonary, Critical Care and Sleep Medicine & Section of Transfusion Medicine and Therapeutic Pathology, University of New Mexico School of Medicine, Albuquerque, USA; 10 Department of Medicine, Sinai Health System and University Health Network, Toronto, Canada; 11 Medical Clinic V, Nephrology, Rheumatology, Blood Purification, Academic Teaching Hospital Braunschweig, Brunswick, Germany; 12 Department of Intensive Care Medicine and Anesthesiology, “Fondazione Policlinico Universitario Agostino Gemelli IRCCS” Università Cattolica del Sacro Cuore, Rome, Italy; 13 Apheresis Unit, Saint-Louis Hospital, AP-HP, University of Paris Cite, Paris, France; 14 Department I of Internal Medicine, Faculty of Medicine and University Hospital Cologne, Center for Integrated Oncology Aachen Bonn Cologne Duesseldorf (CIO), University of Cologne, Cologne, Germany; 15 Apheresis and Cellular Therapy Unit, Department of Hemotherapy and Hemostasis, ICMHO, Clínic Barcelona, IDIBAPS, University of Barcelona, Barcelona, Spain; 16 IRCCS per Oncologia e Neuroscienze, Genoa, Italy; 17 Dipartimento di Scienze Chirurgiche Diagnostiche ed Integrate, Universita’ di Genova, Genoa, Italy; 18 Department of Intensive Care Medicine, Multidisciplinary Intensive Care Research Organization (MICRO), St. James’s Hospital, Dublin, D08 NHY1 Ireland; 19 Department of Clinical Medicine, School of Medicine, Trinity College Dublin, Dublin, D02 PN91 Ireland; 20 Institut D’Investigacions Biomediques August Pi i Sunyer (IDIBAPS), Hospital Clinic, Universidad de Barcelona, Ciberes, Barcelona, Spain; 21 Department of Intensive Care, Guy’s & St Thomas’ Hospital, King’s College London, London, UK; 22 Department of Intensive Care, OLVG Hospital, Amsterdam, The Netherlands; 23 Laboratory of Translational Intensive Care, Erasmus MC, Rotterdam, The Netherlands

**Keywords:** Knowledge, Gaps, Plasmapheresis, Expert panel, Apheresis, Research, Plasma exchange, Research

## Abstract

Therapeutic plasma exchange (TPE) is a therapeutic intervention that separates plasma from blood cells to remove pathological factors or to replenish deficient factors. The use of TPE is increasing over the last decades. However, despite a good theoretical rationale and biological plausibility for TPE as a therapy for numerous diseases or syndromes associated with critical illness, TPE in the intensive care unit (ICU) setting has not been studied extensively. A group of eighteen experts around the globe from different clinical backgrounds used a modified Delphi method to phrase key research questions related to “TPE in the critically ill patient”. These questions focused on: (1) the pathophysiological role of the removal and replacement process, (2) optimal timing of treatment, (3) dosing and treatment regimes, (4) risk–benefit assumptions and (5) novel indications in need of exploration. For all five topics, the current understanding as well as gaps in knowledge and future directions were assessed. The content should stimulate future research in the field and novel clinical applications.

**Supplementary Information:**

The online version contains supplementary material available at 10.1186/s40635-023-00510-w.

## Background

Therapeutic plasma exchange (TPE) is a routine method that separates plasma from blood cells to remove pathological factors or to deliver deficient ones. Consistently, it is used for numerous diseases characterized by the presence of harmful circulating factors or the deficiency of protective components. Given a biological plausibility of benefit in many critical care syndromes [1], it is surprising that there is remarkably little evidence concerning to potential indications, dosing, timing, regimen, and adverse events in the critically ill patient. Given the huge potential of plasma exchange, a recent review has summarized its use in the ICU setting [1]. Here, a group of experts from mixed medical backgrounds used a modified Delphi method to identify remaining gaps in knowledge and framed key research questions for studies on the use of TPE in critically ill patients.

## Methods

**Fig. 1:**
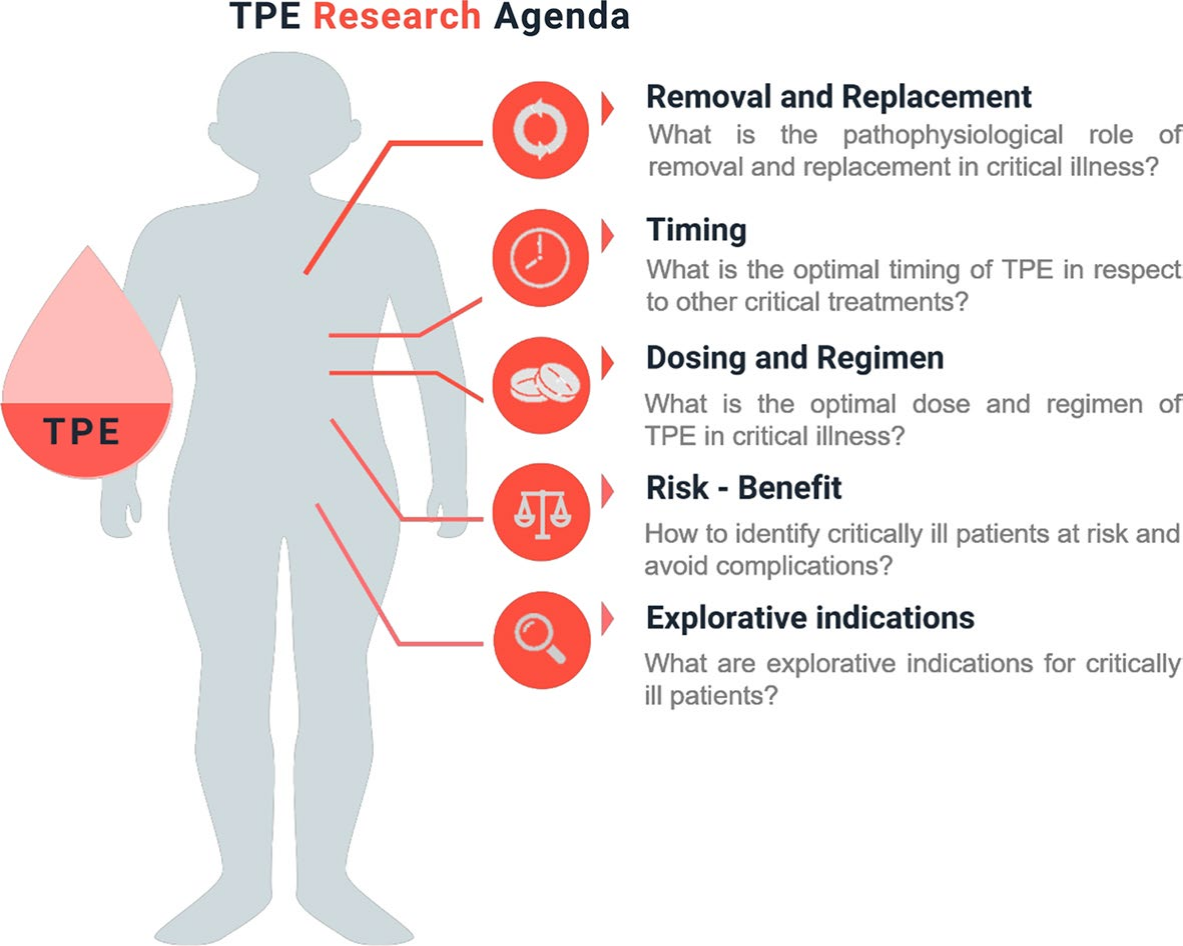
Schematic illustration of key research questions addressed in this article. *TPE* therapeutic plasma exchange

This research agenda was developed in a stepwise approach starting with the development of a panel of 18 international experts from different fields (intensive care medicine, hematology/oncology, nephrology, transfusion medicine Additional file 1: Table S1). An initial list of potential key research questions yielded 16 potential questions. In a next step, 16 of the experts from the panel scored each question from 0 to 5 using an electronic survey tool (Table 1). The five topics with the highest rankings were considered top research priorities and have been addressed in this article (Fig. 1). Each topic was elaborated in a sub-panel of 5 to 7 experts in a structured standardized fashion summarizing both the current knowledge and the knowledge gaps.

**Table 1:** Rating of importance of research questions for plasma exchange in the critically ill patient by sixteen experts in the field

Please score any question q1-16 with a score from 1 to 5 (1 being of little importance for your ideal research agenda, 5 being of very high importance)	Results from 16 experts				
	Mean	Median	SD	IQR	Rank
Q1: What are explorative indications worthwhile studying in the critically ill and/or currently under investigation?	4.8	5.0	0.55	(5–5)	1
Q2: Which inflammatory conditions are indication for TPE?	2.1	2.0	1.20	(1–3)	12
Q3: Which (novel) biomarkers can serve as an indication for benefit of TPE?	2.4	3.0	1.33	(1–3)	10
Q4: Which is the optimal timing of TPE in respect to other treatments (i.e. IV Ig, Rituximab, Cyclophosphamid)	3.7	4.0	1.18	(3–5)	3
Q5: Along the lines of timing- When is it just too late to consider TPE – are there conditions where TPE may be of detriment to recovery if not initiated in the correct time frame e.g. ALF/ACLF?	3.0	3.0	1.08	(2.5–3)	7
Q6: What is the optimal “dose” and regimen” of PE?	3.5	4.0	0.97	(3–4)	4
Q7: Is the exchange solution really important in PE? Which one is the best?	2.2	2.0	1.01	(1–3)	9
Q8: Is central line placement a mandatory requirement to perform plasma exchange in ICU setting?	1.2	1.0	0.55	(1–1)	14
Q9: How to identify patients at risk and to avoid of complications (risk—benefit)	3.8	4.0	1.01	(3–5)	2
Q10: What is the effect of unselective removal of plasma components that might be physiologically upregulated in critically illness (or part of our treatment)? Is re-balancing to “normal” really a good thing if a patient is sick	3.4	4.0	0.87	(3–4)	5
Q11: What kind of antibiotics and other drugs are removed by TPE?.. (including TDM)	2.9	3.0	0.86	(2–3)	6
Q12: Are there interactions between antibiotics and other drugs removed by TPE?	1.9	2.0	0.86	(1–3)	13
Q13: Is there any favorite immunomodulator/immunosuppressant to combine with PE?	2.4	3.0	1.12	(1–3)	10
Q14: Is there a role for micronutrient and vitamin supplementation during treatment with PE?	1.9	2.0	0.76	(1–2.5)	11
Q15: How can we establish the essential educational milestones in apheresis training for ICU specialists?	2.5	2.0	1.20	(1.5–3)	8
Q16 How best to diagnose sepsis in patients receiving PE (when inflammatory markers may be misleading)?	3.3	4.0	1.11	(2.5–4)	5

*TPE* therapeutic plasma exchange, *PE* plasma exchange, *ALF* acute liver failure, *ACLF* acute on chronic liver failure, *ICU* intensive care unit, *TDM* therapeutic drug monitoring, *SD* standard deviation, *IQR* interquartile range

## Results

### Question 1. What is the pathophysiological role of removal and/or replacement of circulating factors during critical illness?

**Fig. 2:**
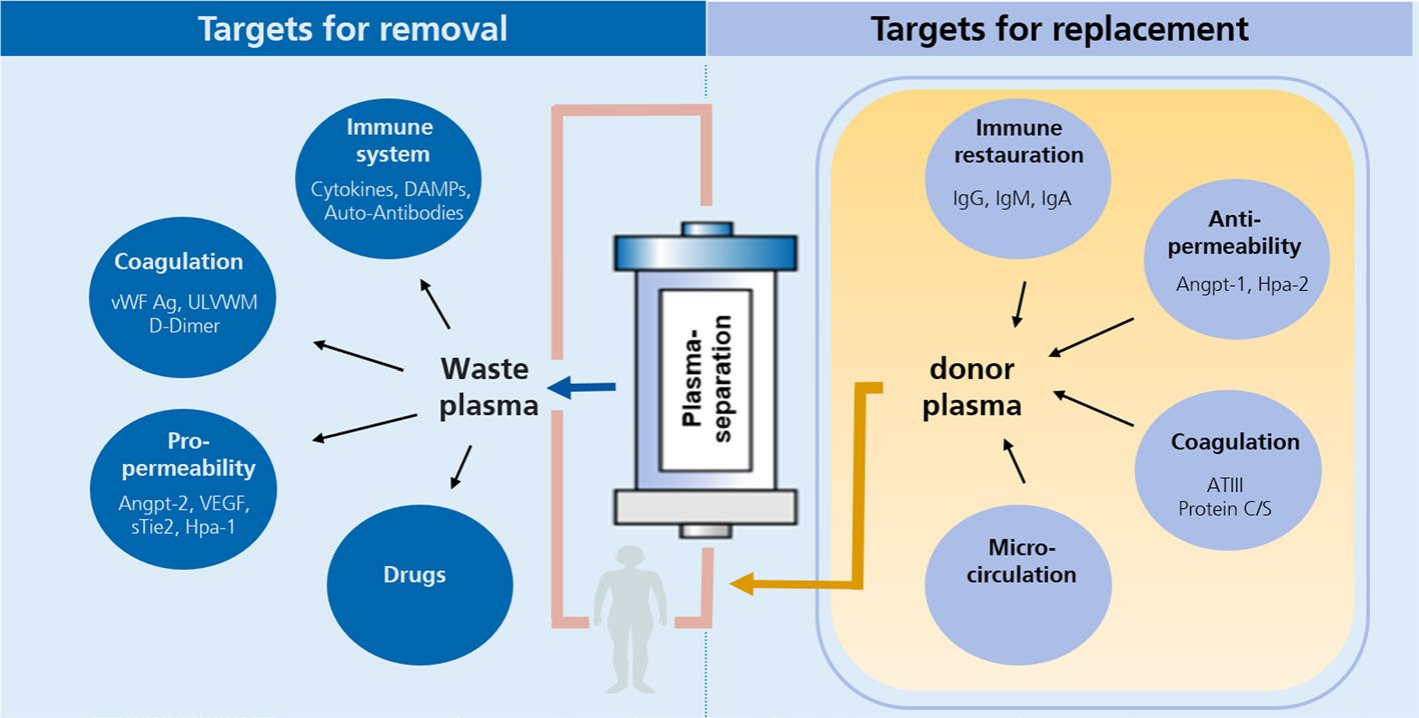
Schematic illustration of potential (explorative) targets for replacement and removal by plasma exchange (adjusted from [99]). *DAMPs* damage associated molecular patterns, *VWF* von Willebrand factor, *ULVWM* ultralarge von Willebrand multimers, *Angpt-1/-2* angiopoietin, *VEGF* vascular endothelial growth factor, *Hpa-1/-2* heparanase, *ATIII* antithrombin III, *ADAMTS13* A disintegrin and metalloproteinase with a thrombospondin type 1 motif, member 13

Filling in the knowledge gaps about the pathophysiologic concepts of TPE, particularly in explorative settings, is important. Increased circulating levels of a molecule that is typically thought injurious might be part of a crucial compensatory mechanism and its removal may harm certain patients. Furthermore, it is important to recognize that TPE combines two procedures in one (i.e.: the removal of potentially harmful plasma components and the replacement of potentially protective ones, Fig. 2).

#### What is known

Both the removal and replacement of factors are dependent on the exchange fluid being used (plasma vs. albumin), the volume replaced, and the kinetics and dynamics of the individual target factor (such as protein binding, volume of distribution). Table 2 summarizes injurious circulating factors that are elevated and/or protective factors that are depleted during various conditions. A prototype of this “replacement” aspect is the vWF-cleaving protease termed “A disintegrin and metalloproteinase with a thrombospondin type 1 motif, member 13” (ADAMTS13) deficiency [2]. Lack of this protease is the characteristic feature of thrombotic thrombocytopenic purpura (TTP) for which TPE is still considered the gold standard treatment [3]. Obviously, in any type of disease, deficient plasma components can only be supplemented if plasma from healthy donors is used as a replacement solution.

**Table 2:** Circulating factors that might be modulated by therapeutic plasma exchange

Removal			
Potential target	Role	Disease	Available data
Cytokines	Inflammation	Sepsis, SIRS	Experimental
Autoantibodies (e.g. ANCA)	Autoimmune	vasculitis, Goodpasture’s syndrome	SOC
Donor-specific antibodies	Rejection	Transplantation	SOC, expert opinion
Immunoglobulins	Hyperviscosity	Hyperviscosity syndrome	SOC
Angiopoietin-2	Permeability	ARDS, sepsis	OBS
vWF antigen	Coagulopathy	Sepsis, DIC	OBS
Heparanase-1	Glycocalyx shedding	Systemic inflammation, Covid-19	OBS
Active viral particles (HSV, EBV)	infectious diseases	Virus-induced acute liver failure	CR
Heparin/PF4 antibody	Coagulation	Heparin-induced thrombocytopenia	CS

Replacement			
Potential target	Role	Disease	Available data
ADAMTS13	vWF cleaving protease	TTP	RCT, SOC
Heparanase-2	Glycocalyx stabilisation	Systemic inflammation, Covid-19	OBS
Immunoglobulins	Ig deficiencies	Infection	OBS
Angiopoietin-1	Anti-permeability	Sepsis, systemic inflammation	OBS
Protein C	Coagulation, microcirculation	Sepsis, purpura fulminans	OBS
Coagulation factors	Coagulopathy/DIC	Investigated in acute liver failure	RCT

*ANCA* anti-neutrophil cytoplasmic antibody, *vWF* von Willebrand factor, *PF4* platelet factor4, *SIRS* systemic inflammatory response syndrome, *ARDS* acute respiratory distress syndrome, *DIC* disseminated intravascular coagulopathy, *HSV* herpes simplex virus, *EBV* Epstein–Barr virus, *ADAMTS13* A disintegrin and metalloproteinase with a thrombospondin type 1 motif, member13; *TTP* thrombotic thrombocytopenic purpura, *SOC* standard of care, *OBS* observational study, *CR* case report, *CS* case series, *RCT* randomized controlled trial

#### What is not known

- What is the effect of TPE on host immune response?

Most studies investigating TPE have shown a decrease in pro-inflammatory cytokines after TPE in a broad range of diseases, such as Systemic Lupus Erythematosus (SLE) and Chimeric Antigen Receptor (CAR) T-cells associated cytokine release syndrome (CRS) [4–8]. Conversely, the anti-inflammatory cytokine IL-10 increased after TPE in patients with myasthenia gravis [7].

However, a fundamental question behind the concept of removal is the consideration that it might not be beneficial per se to remove “bad things”. This concept is of interest for any blood purification technique and refers to the fact that even a massive elevation of an injurious mediator might be a protective strategy of the organism in the sense of an evolutionary preserved compensatory mechanism in a given disease state. In this case, pure extracorporeal removal could be harmful as it has recently been demonstrated for some adsorptive techniques [9].

Another known phenomenon is the “rebound” that occurs after completion of TPE sessions, suggesting a transient effect on cytokines [10] or a re-distribution from third spaces. De novo production of cytokines may also greatly vary [11] and influence plasma cytokine levels according to the underlying disease and the degree of extracorporeal removal. Along these lines, it remains uncertain how the removal of systemically elevated cytokines can modulate their levels within organs and tissues.

Beyond the mechanical removal of circulating antibodies, TPE might be able to indirectly modulate cellular host immune responses as well. TPE has been associated with a decline in B-cell and T-cell activation [5]. However, the potential impact of TPE on skewing T helper (Th) 1/Th2 balance is controversial [6, 12].

Although the clinical consequences remain uncertain to date, the risk of procedure-associated infection appears to be very low [13, 14].

- How to predict the effect of TPE on individual coagulation factors?

TPE results in a ‘regression to the mean’ of circulating proteins, in which coagulation factors that are lacking will increase following exchange with normal donor plasma, whereas factors that are elevated might be diluted to a “normal” level following TPE. As there is significant heterogeneity of individual coagulation factors among healthy donors, the effect of TPE with single donor plasma is hard to predict [11, 15]. Studies that compared TPE sessions using single donor versus pooled plasma (where the anticoagulation factor profile is known and relatively stable) are lacking.

Dilutional coagulopathy occurs when the patients’ plasma is exchanged with albumin or saline [1, 16]. However, TPE does not remove single factors but leads to a balanced reduction of pro- and anticoagulant proteins [17], which might explain the relatively low prevalence of bleeding [18]. Nevertheless, there is significant uncertainty with regards to both coagulation monitoring during exchange regimens and the cumulative effect of multiple exchange sessions [19].

- What is the pathophysiological effect of plasma exchange on the endothelium?

Endothelial dysfunction is a cornerstone of several diseases such as sepsis, COVID-19 or preeclampsia [20–22] and may be modulated by TPE. Rheological phenomena provoked by the exchange process are not well understood [23]. Given the surface localization, the dynamics and susceptibility to flow, the endothelial glycocalyx may be shed due to altered shear stress [24] and to altered plasmatic protein composition. On the other hand, some reports connect properties of human plasma and albumin with stabilization or even restoration of glycocalyx components [25]. Antagonistic glycocalyx modulating enzymes termed heparanases (e.g.: Hpa-1 and Hpa-2) are also re-balanced by TPE and might contribute to endothelial health [26, 27].

- What is the effect of the type of replacement fluids on host responses?

Apart from coagulation, the effects of plasma or non-plasma replacement fluids on immune signalling, endothelial function, inflammation, complement activity, etc. remain largely unexplored. If plasma is the selected replacement fluid, additional questions emerge, such as “what is the optimal type of plasma”—for instance, the potentially different, subtle effects of liquid (never-frozen) plasma compared to fresh frozen plasma (FFP) or plasma frozen within 24 h (FP-24), or standard plasma (FFP or FP-24) compared to cryo-poor plasma (CPP). On an even deeper level, variables such as age/sex/ethnicity/immune status of the donor or proteomic/metabolomics/transcriptomic composition of the units and the processing technique [28] may potentially influence the effects of plasma exchange on the recipient beyond that of the intended primary effect of antibody removal or replacement.

### Question 2. What is the optimal timing of TPE with respect to other concomitant treatments?

The timing of TPE might be crucial in terms of both desired therapeutic and undesired off-target effects, which occur by removing concomitantly administered treatments for the underlying disease, for instance intravenous immunoglobulins (IVIG), rituximab, cyclophosphamide, caplacizumab, and antibiotics.

#### What is known

The amount of components that are removed depends on the volume of distribution of the drug, the half-life of the drug in the circulation, and whether the agent is administered immediately before or even during TPE. In general, compounds with a low volume of distribution (< 0.2 L/Kg) or high protein binding (80%) are removed during TPE [29, 30]. A recent manuscript summarized the theoretical removal rates of COVID-19 drugs based on these pharmacokinetic considerations [31].

#### What is not known

- How should drug administration be timed if a critically ill patient is on a TPE regime?

Current information leaflets of literally any drug completely lack dose recommendations for patients receiving TPE. A list of drugs commonly prescribed in the ICU with a high likelihood of removal by TPE—analogous to the recent COVID-19 paper by Shokouhi [31]—is highly desirable.

It is reasonable to administer drugs after a TPE session to avoid unwanted removal. Nevertheless, for large drugs with a long intravascular half-life, it remains unclear if drug removal still occurs during the next session of TPE up to 24 h later. Re-dosing of drugs such as antibiotics should be considered. Therapeutic drug monitoring (TDM) before and after TPE together with the measurement of levels of drugs in the removed plasma might increase knowledge.

- How should established treatments of an underlying disease be timed if a critically ill patients is on a TPE regimen?

The comparison with alternative strategies such as IVIG e.g. in neurological entities for the induction of disease remission is under-studied and it remains unknown if such gold-standard treatments should be given upfront or at the end of a TPE course in the sense of a rescue approach in refractory scenarios.

### Question 3. What is the optimal dose and regimen of TPE?

Most centres perform three to five sessions on a daily or alternate day fashion. In addition, TPE doses (i.e. the exchanged plasma volumes) are undetermined and there is huge heterogeneity in clinical practice. In TTP, stopping criteria are biologically driven including an increase in platelets above a certain threshold. In other conditions, stopping criteria are less clear.

#### What is known

Knowledge about the removal capacities of plasma proteins is based on studies that assessed IgG and IgM levels during the course of multiple types of TPE. Small molecules with a large volume of distribution (e.g. IgG) have a removal of 65–70% after the first session with a significant redistribution following TPE [29]. The removal of large molecules with a small volume of distribution (e.g. IgM) is similar but lacks the subsequent re-distribution. Under steady-state conditions, 3–5 sessions exchanging 1–1.5 plasma volumes are sufficient to remove most molecules to a level below 90% [1]. However, if the substances are predominantly intravascular (e.g.: IgM), even one session with a high exchange volume might be satisfactory and can remove 86 to 92%. Mathematical models to predict the removal kinetics have been computed [29].

#### What is not known

Known removal rates of IgG and IgM have been generated in stable patients and are likely not applicable to critically ill individuals with alterations in distribution volumes, renal function and nutritional status.

Future pharmacokinetic studies must attempt to identify the ideal regimen in terms of the total number of sessions, frequency intervals, and plasma volume to exchange, and these questions must be evaluated in various disease states.

The monitoring of absolute circulating levels of a putative pathophysiological factor to steer the optimal TPE treatment regimen could be an interesting approach to test. In other words, the actual level of an elevated target (e.g. autoimmune antibody) has never been tested as a predictor of TPE response nor as stopping criteria in a longitudinal fashion.

An apheresis registry collecting the type of apheresis procedures performed in the ICU as well as indications, intensity of therapeutic procedures, reasons to continue or discontinue and associated outcomes is strongly encouraged.

### Question 4. How to identify critically ill patients at risk of complications?

#### What is known

TPE is associated with several complications and undesirable effects. Besides removal of beneficial molecules (discussed earlier), technical complications of TPE comprise catheter- and procedure-related events. Potentially life-threatening complications are rare. The most important ones are anaphylactic reactions occurring in < 0.1 up to 2% of patients [14, 32]. Hypocalcemia is often observed due to a combination of citrate-based TPE circuit anticoagulation and the addition of citrate to plasma as anticoagulant. TPE may cause or worsen bleeding in susceptible patients if the exchange is not performed with plasma [33]. Nevertheless, the incidence remains low (from 0.5 to 5.4%) [34] and most bleeding events are related to central vascular catheter complications. Other complications relate to misinterpretation of routine diagnostic biomarkers as these may also be removed during TPE. An observational study showed that a single TPE reduced the plasma levels of N-terminal (NT)-pro b-type natriuretic peptide (BNP) (NT-pro BNP) by 23%, C-reactive protein (CRP) by 64%, procalcitonin by 31% and troponin-T by 14% [35].

### What is not known

- What factors are removed unintentionally?

So far, no studies have investigated undesired off-target effects of TPE as a results of unintentional removal, particularly not in critically ill patients with severely altered volumes of distribution. Along these lines, data on drug removal by TPE are scarce and mostly based on case reports or series; only a few pharmacokinetic studies have been performed [36–38], and for most drugs, there is no information available. Although kinetic models for the prediction of substance removal have been developed, drug dosing in TPE remains challenging and requires further research.

- How to prevent allergic reactions and deal with re-exposure?

The use of premedication regimens might reduce allergic reactions in certain patient groups [39], but it remains unclear whether pre-medication (and which) should be routinely administered. Within different centers, the standard procedure varies substantially from no pretreatment to antihistamines alone or in combination with steroids. In addition, data on timing and standardized dosing remains scant. Appropriate strategies for re-exposure to FFP following anaphylactic type reactions vary and proposals for standardized investigations to determine risk factors for allergic reactions in the context of TPE are lacking.

- How to reduce the bleeding risk and to dose anticoagulation?

Guidelines from the *American Society For Apheresis* (ASFA) recommend (based on weak evidence) that physicians consider replacing coagulation factors in patients receiving daily TPE with albumin [40]. Specific therapeutic strategies to prevent bleeding in high-risk patients are not well defined. Also, the need for anticoagulation is not completely elucidated. In a study of 367 TPE sessions it appeared that TPE without anticoagulation may be safe [34], although this strategy has not been adopted in routine clinical practice.

- How to interpret routine biomarkers during TPE?

The interpretation of any circulating biomarker during TPE is challenging. For instance, in patients with an infection, a decrease in CRP is usually interpreted as disease resolution. If a patient is simultaneously receiving TPE daily regimen, such a fall in CRP cannot be attributed only to resolving infection but may also be due to removal during TPE. These considerations are valid for any marker circulating in plasma (e.g.: liver enzymes, renal test, cardiac biomarker, etc. [35]) the degree of removal of these biological markers during TPE is unknown. The most effective strategies to monitor organ function during TPE and recognize complications are not fully understood. Studies that analyze the elimination rate of routine biomarkers by TPE are highly desirable.

## Question 5. What are explorative TPE indications for critically ill patients?

In theory, any condition induced by a known or suspected circulating factor might benefit from its removal. Diseases and syndromes such as sepsis, hemophagocytic lymphohistiocytosis (HLH), CAR T-cell associated cytokine release syndrome (CRS), and pancreatitis share common phenotypic features of systemic hyper-inflammation with endothelial dysfunction and coagulopathy. The excess release of injurious cytokines, damage-associated molecular patterns (DAMPs), cell-free DNA and neutrophil extracellular traps (NETs) together with a reduction in protective plasmatic factors are involved in many pathophysiological processes. In this section, we discuss the potential of TPE to improve some of these syndromes (structured in seven core topics summarized in Additional file 1: Table S2.

### Systemic inflammatory conditions

#### What is known

In sepsis, TPE might modulate biological efficacy endpoints related to inflammation, coagulation, microcirculation and endothelial function [27, 41, 42]. A recent pilot randomized controlled trial (RCT) in early refractory septic shock demonstrated improved hemodynamics within 6 h compared to standard of care [43] and a propensity score matched analysis [44] revealed a potential survival benefit. Mechanistically, it has been shown that restoration of the balance between reduced ADAMTS13 activity and elevated VWF-antigen to a normal ratio might improve microvascular perfusion and flow. Along the same line, TPE can replace consumed anticoagulant proteins such as Protein C [42].

TPE has also been proposed as a therapeutic option to treat critically ill COVID-19 patients [45]. A recent systematic review of 267 patients demonstrated safety and some biological efficacy with regards to inflammation and immune activation [46]. An RCT of 87 patients with life-threatening COVID-19 found a shorter ventilation time and even a trend towards lower mortality (20.9 vs. 34.1%) [47].

Data on TPE in sterile systemic inflammatory conditions such as acute pancreatitis, CAR T-cell associated CRS, HLH or macrophage activation syndrome (MAS) etc. are mostly limited to case series [48–52].

#### What is not known

- Is TPE associated with a positive clinical effect?

We still fail to comprehensively monitor the immune system confronting us with a situation where some patients might benefit from the removal of inflammatory mediators while others could suffer harm by the same intervention. Besides a theoretical rationale and an increasing body of evidence regarding biological and some efficacy endpoints, it remains unclear whether improvements in surrogates such as circulating mediators actually translate into improved clinical conditions.

- Which inflammatory patient would benefit?

In terms of response prediction, it has been reported that baseline lactate levels were predictive of the effect of TPE on hemodynamic stabilization during the initial 24 h [42]. Given the role of lactate in states of microcirculatory compromise, it can be speculated that systemically inflamed patients with microvascular coagulopathy might be more susceptible to TPE (which might close the loop to the ADAMTS13/vWF axis as mentioned earlier).

### Trauma, thermal injury, burns

#### What is known

Trauma and burns can result in strong pro-inflammatory host responses mostly driven by enormous amounts of circulating DAMPs with ensuing endothelial hyper-permeability and organ failure. Circulating levels of endothelial glycocalyx constituents in trauma are associated with adverse outcome [53].

Despite increasing recognition of the pleiotropic effects of plasma in bleeding trauma patients and the fact that plasma transfusion can improve outcomes [54–56], there are no data on the use of TPE in critically ill trauma patients. In a limited number of burn patients, TPE as a rescue intervention was associated with rapid resolution of shock parameters [57, 58].

#### What is not known

- Could plasma exchange modulate the injurious host response to severe trauma?

As alluded to above, pleiotropic effects of plasma have been studied increasingly but the procedure of TPE per se has been neglected so far. In general, it is also uncertain whether plasma is beneficial or just that the comparator fluid (i.e. crystalloids) is detrimental, as dilution of the protein content following clear fluid infusion may result in shedding of the glycocalyx. In line with this thought, it is not known whether merely improving protein content is beneficial or whether there are specific repair factors in plasma. The ADAMTS13/vWF imbalance in sepsis also exists in patients with trauma-induced organ injury [59]. Also, it is unknown whether removal of compounds such as DAMPs with TPE is superior over plasma transfusion alone.

Severe capillary leakage is a common phenomenon in severe burn patients. In other inflammatory conditions, it has been demonstrated that TPE might be able to modulate endothelial permeability rebalancing various systems such as glycocalyx, Angiopoietin/Tie2 and potentially also bioactive adrenomedullin (bio-ADM). These observations make TPE a desirable tool to study in severely burned patients [60].

### Coagulopathies (HIT and DIC)

#### What is known

Heparin-induced thrombocytopenia (HIT) is a pro-thrombotic complication of heparin where platelet-activating antibodies develop after exposure to heparin leading to thrombosis and thrombocytopenia [61]. TPE has been described for refractory HIT conditions to remove such specific antibodies [62].

TPE has also repeatedly been reported as a therapeutic option in sepsis-mediated disseminated intravascular coagulopathy (DIC). A recent prospective study found improved platelet counts, coagulation function and even increased survival rates after TPE [63] which is in line with observations in patients with organ failure, DIC, meningococcal septicemia, *Capnocytophaga canimorsus* infection and scorpion bites [63–66].

#### What is not known

- What is the specific molecular target of TPE in DIC patients?

Despite the rationale to rebalance a disturbed coagulation system, in DIC, the exact target molecule is unknown. Potential candidates may be NETs and DAMPs [67], removal of which might result in an improvement of DIC. As the degree of hypofibrinolysis may vary depending on the underlying condition [68, 69], it is possible that certain subtypes of DIC where the suppression of the fibrinolytic system dominates will be more responsive to TPE. Altogether, TPE will most likely remove D-Dimers and alter fibrinogen levels but will not directly affect thrombocytopenia.

### Neurological conditions

#### What is known

Improved recognition of rapidly progressive severe antibody-mediated central nervous system disorders, has led to an increasing interest in the use of TPE for a variety of different neurological disorders [1].

The main potential indications include antibody-mediated central nervous system (CNS) disorders such as anti- N-methyl-D-aspartate (NMDA) receptor encephalitis [70], myasthenia gravis and Guillain-Barré syndrome (GBS) [70–72]. Most of the experience however is limited to case series in multiple sclerosis and neuromyelitis optical spectrum disorders [73].

#### What is not known

- Are there therapeutic effects of TPE in neuro-immunological diseases that go beyond the sole removal of antibodies?

In autoimmune encephalitis, the underlying mechanism of the benefit of TPE seems to be more complex than simply removing circulating pathogenic antibodies. Removal of immune complexes and cytokines or modification of the representation and function of regulatory T-cells (T_reg_) and natural killer cells [3] might also be beneficial.

- Is there a role for TPE in the maintenance of disease remission?

Further research is needed to determine the role of long- term treatment effects and also TPE regimens as maintenance strategies. Overall, current TPE regimens with regard to duration, frequency as well as cessation criteria are largely arbitrary and there is huge variation between individual centers. In general, this holds true for established and explorative TPE indications, too, and is not limited to neurological entities.

### Non-hematological (auto)immune conditions

#### What is known

While TPE has an already established role in certain autoimmune diseases, its role in non-hematological autoimmune disorders and rheumatic diseases is less clear [74].

Based on a negative RCT in patients with systemic lupus erythematosus (SLE) and nephritis [75], TPE is not recommended by the current ASFA guidelines [76]. In renal crisis due to systemic sclerosis, a single-center observational study reported better outcomes in patients where a combination therapy of TPE and angiotensin-converting-enzyme inhibitors was used [77]. In severe ANCA-associated vasculitis (AAV) a benefit of TPE to standard immunosuppressive regimens could not be demonstrated [78]. Nevertheless, these patients were not necessarily critical ill and did not suffer from diffuse alveolar hemorrhage. In patients with thyroid storm, TPE was shown to be effective in a small case series, in particular for those with poor response to conventional standard therapies [79, 80]. Obviously, the mode of action in thyrotoxicosis lies in the ability to remove thyroid hormones [81].

#### What is not known

- What is the differential response at the individual organ level in systemic diseases involving multiple organs?

Apart from nephritis, the role of TPE in SLE with serious organ involvement, such as diffuse alveolar hemorrhage (DAH), pulmonary capillaritis [71, 82, 83], neuropsychiatric involvement [84] or myocarditis is not clear. Evidence provided by small case series and reports support a potential benefit in this context [85].

With regard to systemic sclerosis, despite the aforementioned potential role in severe renal crisis, the role of TPE in other forms of systemic sclerosis, such as diffuse skin sclerosis is unclear [86]. Given the rationale that the pathogenesis of skin sclerosis is closely related to circulating factors, some authors recommend the use of TPE in these situations [87]. However, the literature is mostly outdated and does not reflect modern techniques. Another limitation is based on the heterogeneity of study design in which a wide range of adjunctive strategies were administered along with TPE [88, 89].

- When to start TPE in (refractory) thyroid storm?

Despite promising data suggesting that TPE could be an effective therapy in patients with thyroid storm, the exact criteria for initiation, i.e. severity or clinical manifestations are unclear. In particular, it remains unknown whether TPE should be used as a first line therapy for severe manifestations or be reserved for refractory situations when traditional treatments have failed [81].

### Transplantation

#### What is known

TPE has an established role in transplantation, both prior and afterwards. The transplant recipient`s defense mechanisms can be triggered in numerous ways of which some build the biological plausibility for the use of TPE.

TPE is implemented in desensitization protocols for ABO-incompatible (ABOi) transplantation to lower the isoagglutinin titers and has been used to mitigate hyperacute rejection mediated by class 1 antigens [90, 91]. TPE has also been used to remove so-called donor specific antibodies (DSA) to treat humoral rejection [92–94]. Transplant-associated thrombotic microangiopathy is a complication for which complement inhibition has emerged as a treatment of choice, but in situations where this approach is ineffective, TPE could be used as an alternative adjunctive strategy [95, 96].

TPE in addition to immunosuppression has been reported to show reasonable success in inducing remission of recurrent autoimmune disease in a successfully transplanted graft [97, 98].

#### What is not known

- What are the ideal pre- and post- transplant TPE protocols?

TPE is included in numerous pre- and post-transplant protocols as standard of care, mostly because of the biological plausibility of the concept. However, the best frequency, timing of initiation, dosing and modality remain unclear due to lack of standardization in practice and controlled trials.

- Are all antibody-mediated rejections (AMR) the same or is there a hierarchy of clinical significance of certain Donor Specific Antibodies (DSA) that could help to guide individual TPE strategies?

The close interplay between cellular (T-cell mediated) and humoral rejection is increasingly being recognized [93]. The underlying reasons for TPE refractoriness are not clear and a systematic histological analysis may offer some additional clues to group AMR patients from a tissue phenotype point of view to avoid the unnecessary use of TPE.

The correlation between antibody levels and onset of immunological phenomena such as AMR is not straightforward. Whether the risk is linear or whether additional factors play a cumulative role is not delineated. Hence, the goal of TPE in this context remains open for debate. In other words, it is unclear whether DSA concentrations (i.e. mean fluorescence intensity (MFI)) and classes (HLA type 1 vs type 2) should determine the intensity and duration of treatments.

### Future directions

As highlighted in this manuscript, there are many promising areas with a strong biological plausibility for the use of TPE but most of the evidence suggesting a potential benefit in the context of explorative indications stems from case reports, small case-series, and retrospective or uncontrolled prospective data. There is an urgent need for trials to justify the clinical application of TPE in these settings. However, given the low incidence of some of the diseases discussed, appropriately powered trials are only feasible as a team effort with international collaboration. There are also relevant concerns about unwanted off-target effects, and uncertainties in dosing and timing.

Both observational and interventional studies are needed to determine parameters (i.e., imaging, biomarkers) to identify patients likely to benefit from TPE and those who may come to harm. Along the same lines, reliable stopping criteria are highly desirable. A development of a TPE registry would facilitate better understanding of the challenges in rarer conditions and possibly highlight any differences based on demographics.

Longitudinal bio-banking data collection could help to analyse the effect of TPE on host immune responses. Regarding the simultaneous removal of circulating biomarkers, alternative tools to assess resolution of infection are needed along with such TPE-driven changes in humoral inflammatory parameters (such as CRP or PCT).

Therapeutic drug monitoring (TDM) with determination of antibiotic levels and other essential drugs before and after treatment as well as in the apheresis waste plasma could provide novel pharmacokinetic insights, especially for drugs necessary to treat the underlying illness, such as immunosuppressants, monoclonal antibodies and anti-microbial agents. Using this information to develop a simple tool to support clinicians in predicting the impact of TPE on drug levels in individual patients should be an essential goal.

More systematic multicentric standardized data collection to evaluate the risk of allergic reactions should be encouraged to evaluate risk factors related to underlying disease, patient and choice of replacement fluid, anticoagulation, circuit, etc. Head-to-head comparisons of the various types of plasma may also be useful to understand the immunological basis for the adverse reactions. We also need prospective studies evaluating the effects of the replacement fluid (albumin versus plasma) and anticoagulant use on bleeding complications during the TPE procedure, thereby working towards defining specific preventive strategies in high-risk patients.

Two multicenter sepsis trials both in Europe and Canada (NCT05093075) will commence in 2023 investigating the role of TPE in systemic inflammatory conditions. Also, in severe COVID-19, several RCTs are still recruiting (e.g.: NCT04685655). On the other hand, syndromes with less heterogeneity than sepsis (e.g.: CAR-T associated CRS) might be easier to study. Particularly, in neurological critical care where the discovery of novel characteristic antibodies has been accelerating, a progressive increase in the number of clinical TPE applications can be expected but will need further guidance from clinical trials. Besides the known role of plasma application in the trauma patient, the exchange of plasma has never been evaluated in trauma-associated organ failure opening potential avenues for a novel approach. The role of TPE in the whole field of autoimmunity and transplantation seems more established but evidence is not much stronger.

Not all knowledge gaps can be answered in such observational clinical studies. Some molecular questions, e.g.: on the endothelial microcirculatory environment are best studied in animal disease models.

Finally, future studies must include patients' views to ensure that patient-centered outcomes are evaluated and the benefit-risk balance is assessed.

### Take home message

The use of therapeutic plasma exchange in the critically ill patient is increasing, but the biological concept of simultaneous removal of injurious and replacement of protective molecules is double-edged and requires future research. A group of experts around the globe elaborated on key research questions focusing on pathophysiology, timing, dosing, side effects and explorative indications.

## Supplementary Information

**Additional file 1: Table S1**. Expert panel composition.** Table S2**. ASFA recommendations for explorative indicationsadopted from Schwartz et al. [76].

## Data Availability

Not applicable.

## Abbreviations

TPE: Therapeutic plasma exchange
ICU: Intensive care unit
VWF: Von Willebrand factor
ADAMTS13: A disintegrin and metalloproteinase with a thrombospondin type 1 motif, member 13
TTP: Thrombotic thrombocytopenic purpura
SLE: Systemic Lupus Erythematosus
CAR: Chimeric Antigen Receptor
CRS: Cytokine release syndrome
COVID-19: Coronavirus disease 2019
Hpa: Heparanase
FFP: Fresh frozen plasma
CPP: Cryo-poor plasma
IVIG: Intravenous immunoglobulins
TDM: Therapeutic drug monitoring
NT-proBNP: N-terminal (NT)-pro b-type natriuretic peptide (BNP)
CRP: C-reactive protein
PCT: Procalcitonin
TropT: Troponin-T
ASFA: American Society For Apheresis
HLH: Hemophagocytic lymphohistiocytosis
DAMPs: Damage-associated molecular patterns
NETs: Neutrophil extracellular traps
RCT: Randomized controlled trial
MAS: Macrophage activation syndrome
Bio-ADM: Bioactive adrenomedullin
HIT: Heparin-induced thrombocytopenia
DIC: Disseminated intravascular coagulopathy
CNS: Central nervous system
NMDA: N-methyl-d-aspartate
GBS: Guillain–Barré syndrome
ACE: Angiotensin-converting-enzyme
DAH: Diffuse alveolar hemorrhage
DSA: Donor specific antibodies
AMR: Antibody-mediated rejection
HLA: Human leucocyte antigen
MFI: Mean fluorescence intensity
ALF: Acute liver failure
ACLF: Acute on chronic liver failure
SD: Standard deviation
IQR: Interquartile range
ANCA: Anti-neutrophil cytoplasmic antibody
PF4: Platelet factor 4
SIRS: Systemic inflammatory response syndrome
ARDS: Acute respiratory distress syndrome
HSV: Herpes simplex virus
EBV: Ebstein barr virus
SOC: Standard of care
OBS: Observational study
CR: Case report
CS: Case series
ULVWM: Ultra-large von Willebrand multimers
Angpt: Angiopoietin
VEGF: Vascular endothelial growth factor
ATIII: Antithrombin III
