# Supplementary material for: Research priorities for therapeutic plasma exchange in critically ill patients
Source: Intensive Care Med Exp. 2023 May 8;11:26. doi: 10.1186/s40635-023-00510-w (PMC10164453; doi:10.1186/s40635-023-00510-w)
Supplement: Supplementary file 1 — Additional file 1: Table S1. Expert panel composition. Table S2. ASFA recommendations for explorative indicationsadopted from Schwartz et al. [76]. [file 40635_2023_510_MOESM1_ESM.docx]

**Table S1.** Expert panel composition (alphabetical order)

| **NAME** | **BACKGROUND** | **INSTITUTION** | **COUNTRY** | **RESEARCH INTEREST** | **CONTACT** |
| --- | --- | --- | --- | --- | --- |
| Anne C Brignier | Hematology, Apheresis | Apheresis Unit, Saint-Louis Hospital, AP-HP, University of Paris Cit | France | Therapeutic apheresis | [anne.brignier@aphp.fr](mailto:anne.brignier@aphp.fr) |
| Pedro Castro | Intensivist, Internist | Hospital Clínic of Barcelona; IDIBAPS; University of Barcelona | Spain | TPE in medical diseases; Critical illness in Immunocompromised Patients | [pcastro@clinic.cat](mailto:pcastro@clinic.cat) |
| Joan Cid | Hematology and Hemotherapy | Apheresis and Cellular Therapy Unit, Department of Hemotherapy and Hemostasis, ICMHO, Clínic Barcelona, IDIBAPS, University of Barcelona | Spain | Therapeutic apheresis | [jcid@clinic.cat](mailto:jcid@clinic.cat) |
| Sascha David | Intensivist, Nephrologist, Internist | University Hospital Zurich, Institute of Intensive Care & Hannover Medica School, Department of Nephrology | Switzerland / Germany | TPE in systemic inflammation | [sascha.david@usz.ch](mailto:sascha.david@usz.ch) |
| Nicole Juffermans | Intensivist Anesthestist | Department of Intensive Care, OLVG Hospital, Amsterdam & Laboratory of Experimental Intensive Care and Anesthesiology | The Netherlands | coagulopathy in trauma patients | [n.p.juffermans@amsterdamumc.nl](mailto:n.p.juffermans@amsterdamumc.nl) |
| Jan T Kielstein | Intensivist, Nephrologist | Brunswick Hospital, Department of Nephrology | Germany | intoxication, blood purification | [kielstein@yahoo.com](mailto:kielstein@yahoo.com) |
| Matthias Kochanek | Intensivist, Hematology and Oncology, Internal medicine | University of Cologne, Faculty of Medicine and University Hospital | Germany | TPE in systemic inflammation | [matthias.kochanek@uk-koeln.de](mailto:matthias.kochanek@uk-koeln.de) |
| Andry Van de Louw | Intensivist | Medical Intensive Care Unit, Penn State Health Hershey Medical Center, Hershey, PA | USA | Critically ill immunocompromised patients | [avandelouw@pennstatehealth.psu.edu](mailto:avandelouw@pennstatehealth.psu.edu) |
| Bruno L Ferreyro | Intensivist, Internist | University of Toronto, Sinai Health System | Canada | Critically ill immunocompromised patients | [bruno.ferreyro@uhn.ca](mailto:bruno.ferreyro@uhn.ca) |
| Marlies Ostermann | Intensivist, Nephrologist, Internist | King's College London, Guy's & St Thomas' Hospital, London | UK | acute kidney injury, multi-organ failure, extracorporeal organ support | Marlies.Ostermann@gstt.nhs.uk |
| Chiara Robba | Intensivist Anesthestist | Policlinico San Martino, Genova | Italy | TPE in neurological diseases | [kiarobba@gmail.com](mailto:kiarobba@gmail.com) |
| Lene Russell | Intensivist, Anesthestist | Dep of Intensive Care, Copenhagen University Hospital Gentofte | Denmark | Thrombocytopenia/coagulopathy, Critical illness in the immunocompromised | [lene.russell@mail.dk](mailto:lene.russell@mail.dk) |
| Eric Mariotte | Intensivist | Medical Intensive Care Unit, Saint-Louis hospital, AP-HP, University of Paris Cité | France | Critical Illness in Immunocompromised Patients | [eric.mariotte@aphp.fr](mailto:eric.mariotte@aphp.fr) |
| Ignacio Martin-Loeches | Intensivist | Department of Intensive Care Medicine, Multidisciplinary Intensive Care Research Organization (MICRO), St. James's Hospital, Dublin | Ireland / Spain | Sepsis, immunoparalysis, criticall illness in immunocompromised patients | [drmartinloeches@gmail.com](mailto:drmartinloeches@gmail.com) |
| Luca Montini | Intensivist | Department of Intensive Care Medicine and Anesthesiology "Fondazione Policlinico Universitario Agostino Gemelli IRCCS" Università Cattolica del Sacro Cuore, Rome | Italy | Crticall illness in immunocompromised patients | [lucamariamontini@alice.it](mailto:lucamariamontini@alice.it) |
| Nathan D. Nielsen | Intensivist, Transfusion Medicine/Apheresis | University of New Mexico School of Medicine | USA | TPE in sepsis; DIC | [nathan.nielsen@gmail.com](mailto:nathan.nielsen@gmail.com) |
| Tasneem Pirani | Intensivist, hepatologist, Internist | King's College Hospital, General and Liver Intensive Care | UK | TPE in ALF and ACLF | [tasneempirani@nhs.net](mailto:tasneempirani@nhs.net) |
| Lara Zafrani | Intensivist | Medical Intensive Care Unit, Saint-Louis hospital, AP-HP, University of Paris Cité | France | Critical Illness in Immunocompromised Patients | [lara.zafrani@aphp.fr](mailto:lara.zafrani@aphp.fr) |

Abbreviations: TPE (therapeutic plasma exchange), DIC (disseminated intravascular coagulopathy), ALF (acute liver failure), ACLF (acute on chronic liver failure)

**Table S2**. ASFA recommendations for explorative indications (categories III and IV) adopted from Schwartz et al. [76]

| **Categories** | **Disease** | **Rationale** | **No. of reported patients** | **Replacement fluid** |
| --- | --- | --- | --- | --- |
| HEMATOLOGICAL | Amyloidosis, systemic | to manage rapidly progres- sive glomerulonephritis (RPGN) with AA amyloidosis; to reduce serum Interleukin-6 levels | 8 | nn |
|  | Aplastic anemia, pure red cell aplasia | removing autoantibodies and/or soluble inhibitory factor | 47 | Albumin, plasma |
|  | Autoimmune hemolytic anemia; warm autoimmune hemolytic anemia; cold agglutinin disease | remove pathogenic immune complexes, activated complement components, and circulating autoantibodies | 76 | Albumin |
|  | Hematopoietic stem cell transplantation, HLA desensitization | removal of HLA donor-specific antibody | 24 | Albumin |
|  | Hemophagocytic lymphohistiocytosis; Hemophagocytic syndrome; Macrophage activating syndrome | removal of hyperinflammatory cytokines | 35 | Albumin, plasma |
|  | Immune thrombocytopenia | removal of autoantibodies and immune complexes | 32 | nn |
|  | Thrombotic microangiopathy, coagulation mediated | Thrombomodulin mutation | 8 | Plasma |
|  | Thrombotic microangiopathy, complement mediated | removal of autoantibody or mutated circulating complement regulators ± replacing absent or defective complement regulator | 203 | Plasma, albumin |
|  | Thrombotic microangiopathy, hematopoietic stem cell transplantation associated | NN | 363 | Plasma |
|  | Thrombotic microangiopathy, toxin mediated | reduction of various cytokines | 1608 | STEC-HUS: Plasma; pHUS: Albumin |
| NEUROLOGIC | Chronic focal encephalitis (Rasmussen Encephalitis) | remove autoantibodies, against several neural molecule | 14 | Albumin |
|  | Complex regional pain syndrome | remove auto-antibodies to beta-2-adrenergic, alpha-1-adrenergic, and muscarinic M2 receptors (and possibly cytokines) | 42 | Albumin |
|  | Paraneoplastic neurological syndromes | removal of onconeural antibodies | 240 | Albumin |
|  | Paraproteinemic demyelinating neuropathies/chronic acquired demyelinating polyneuropathies | removal anti-MAG antibodies, IgG/ IgA/ IgM | 231 | Albumin |
|  | Stiff-person syndrome | removal of autoantibodies | 44 | Albumin |
|  | Sudden sensorineural hearing loss | removal of autoantibodies | 22 | Albumin |
| RENAL | Anti-glomerular basement membrane disease (Goodpasture’s syndrome) | removing autoantibodies (anti-GBM) | 506 | Albumin; for diffuse alveolar hemorrhage plasma |
|  | Immunoglobulin A nephropathy | removal of circulating pathologic IgA molecules and related immune complexes | 81 | Albumin |
|  | Nephrogenic systemic fibrosis | removal of gadolinium containing contrast agents | 34 | Albumin |
| COAGULOPTHY | Coagulation factor inhibitors | removal of antibodies | 124 | Plasma |
| LIVER | Acute liver failure | remove albumin bound/ unbound toxins; removal of inflammatory mediators | 1411 | Plasma, Albumin |
|  | Acute on chronic liver failure | remove albumin bound/ unbound toxins; removal of inflammatory mediators |  | Plasma |
|  | Erythropoietic porphyria, liver disease | to decrease the protoporphyrin level in plasma | 18 | Albumin, plasma |
|  | HELLP syndrome | removal of circulating protein bound platelet aggregating and procoagulant factors | 118 | Plasma |
|  | Pruritus due to hepatobiliary diseases | removal of potential pruritogen(s) | 13 | Albumin |
| DERMATOLOGY | Atopic (neuro-) dermatitis (atopic eczema), recalcitrant | reduce IgE and immune complexes | 9 | Albumin |
|  | Dermatomyositis/polymyositis | removal of autoantibodies (ANA, anti-Ro, anti-La, anti-Sm, anti-ribonucleoprotein, or myositis-specific antibodies) | 44 | nn |
|  | Pemphigus vulgaris | removal of pathogenic autoantibodies | 140 | Albumin, plasma |
|  | Psoriasis | removal of cytokines and putative “psoriatic factor” | 29 | NN |
|  | Scleroderma (systemic sclerosis) | removal of autoantibodies | 166 | Albumin |
|  | Toxic epidermal necrolysis | removal of drug/drug metabolites, cytokines, or other mediators of keratinocyte cytotoxicity | 137 | Plasma, albumin |
| OTHERS | Burn shock resuscitation | remove circulating factors such as inflammatory mediators or other humoral substances participating in major burn pathophysiology | 185 | Albumin, plasma |
|  | Cardiac neonatal lupus | removal of antibodies | 35 | Albumin |
|  | Henoch-Schönlein purpura | removal of IgA-containing immune complexes or IgG autoantibodies | 85 | Albumin |
|  | Heparin induced thrombocytopenia & thrombosis | removal of antibodies specific for complexes of platelet factor 4 (PF4) and heparin; Pre-cardiopulmonary bypass | 71 | Albumin, plasma |
|  | Hypertriglyceridemic pancreatitis | removal of triglycerides | 294 | Albumin, plasma |
|  | Lung transplantation | removal of antibody | 51 | Albumin |
|  | Overdose, envenomation and poisoning | removal of Amanita mushroom poisoning + various drugs and poisonings | >574 | Albumin, plasma |
|  | Post transfusion purpura | Removal of platelet alloantibodie | 26 | Albumin, plasma |
|  | Red cell alloimmunization in pregnancy | removes maternal RBC alloantibody | 337 | Albumin |
|  | Sepsis with multi-organ failure | removing inflammatory and antifibrinolytic mediators and replenishing anti- coagulant proteins and ADAMTS13 | 591 | Plasma |
|  | Thyroid storm | removal of plasma proteins binding T3 ad T4 | 48 | Plasma, albumin |
| HEART | Dilated cardiomyopathy, idiopathic | removal of autoantibodies of various myocardial antigens (a-Myosin, b1-adrenergic receptor, Troponin-I, Na-K-ATPase, M2-muscarinic acetylcholine receptor) | 10 | Albumin |
